# DGAT‐1 deficiency: Congenital diarrhea and dietary treatment

**DOI:** 10.1002/jpr3.70016

**Published:** 2025-04-09

**Authors:** Clemens Gardemann, Ulrike Och, Manfred Fobker, Thomas Kaiser, Judit Horvath, Beatrice Da Prada, Thorsten Marquardt

**Affiliations:** ^1^ Department of Pediatrics University Hospital Muenster Muenster Germany; ^2^ Centre of Laboratory Medicine University Hospital Muenster Muenster Germany; ^3^ Department of Pediatrics, Gastroenterology University Hospital Muenster Muenster Germany; ^4^ Department of Human Genetics University Hospital Muenster Muenster Germany; ^5^ University of Pavia Pavia Italy

**Keywords:** DGAT‐1, gastroenterology, congenital diarrhea, MCT

## Abstract

DGAT‐1 (Diacylglycerol Acyltransferase‐1) deficiency is an autosomal recessive disorder which causes severe impairment in lipid absorption. We report a case of an infant suffering from persistent diarrhea starting at the age of four weeks. Further investigations identified DGAT‐1 deficiency as underlying cause. A treatment plan was developed which included a very‐low fat diet administered as infant formula, essential fatty acid supplementation, C8 medium chain triglycerides‐ and fat‐soluble vitamin supplementations. The patient was put into full remission after administration of the treatment plan and weight curves normalized at the 50th percentile at the age of 24 months. Intermittent episodes of loose stools were due to an excessive intake of fructose via extensive fruit consumption. DGAT‐1 deficiency is a rare genetic disease which leads to congenital diarrhea and is especially dangerous in infancy. Our treatment plan put the patient into full remission showing that C8 MCT oil should be preferred over treatment with C8/C10 mixtures.

## Abstract (highlights)

**What is Known:**

- DGAT‐1 deficiency causes severe impairment in lipid absorption.
- This leads to persistent diarrhea, which is especially dangerous in infancy.
- Cases reported in the literature used solely fat‐restricted/‐free diets to treat patients.

**What is New:**

- We were able to put the patient into full remission by administering a very low‐fat diet, including caprylic acid (C8) medium‐chain triglyceride (MCT) oil, which led to the normalization of weight percentiles.
- C8 MCT oil did not cause diarrhea in our patient.
- To prevent essential fatty acid deficiency, an omega‐3/6 supplement should be advised.

## 1 INTRODUCTION

### 1.1 Background

DGAT‐1 (Diacylglycerol Acyltransferase‐1) deficiency is an autosomal recessive disorder which was first described in 2012.^1^ Two proteins, DGAT‐1 and DGAT‐2 are responsible for intracellular triglyceride biosynthesis. DGAT converts diacylglycerols to triacylglycerols by adding an acyl‐CoA moiety as final step of triglyceride (TG) synthesis. DGAT‐1 is expressed in multiple tissues in the human body including the mammary gland and adipose tissue, but also in the liver and skin. The highest expression of DGAT‐1 is present in the small intestine. DGAT‐2 has very low expression in the small intestine and has its highest expression in the liver and white adipose tissue.^2^

DGAT‐1 plays a key role in intracellular lipid biosynthesis in the small intestine. Dietary fat is cleaved to fatty acids and glycerol in the lumen of the small intestine. These components are taken up by the gut epithelial cells, that will re‐synthesize triglycerides for transport via chylomicrons. DGAT‐1 deficiency leads to an intracellular diacylglycerol accumulation and lipid malabsorption.^3^ Humans with loss‐of‐function of DGAT‐1 suffer from severe congenital diarrhea.^4^ An investigation of 10 patients with DGAT‐1 mutations lead to the conclusion that a fat‐free diet might be the first line of treatment.^5^

### 1.2 Animal models and possible implications for humans

Loss of function of DGAT‐1 in mice leads to decreased body weight, increased energy expenditure and resistance to diet induced obesity.^6^, ^7^ These mice show increased longevity.^8^ Both, DGAT ‐1 and ‐2 inhibition, in mice causes a protective effect for their hearts when given a high fat diet without adverse effects.^9^ Both inhibition and genetic deletion of DGAT‐1 reduced postprandial TG, delayed gastric emptying and inhibited chylomicron formation.^10^

As mice lacking DGAT‐1 show resistance to weight gain and improved insulin sensitivity, the enzyme has been proposed as potential target for treating obesity and obesity‐related illnesses in humans. Several compounds have been tested to inhibit DGAT‐1 in mice and show promising results.^11^ One trial showed administration of DGAT‐1 inhibitors to humans resulted in dose dependent, intolerable side effects, mostly diarrhea.^12^ Several companies therefore stopped their phase one trials for further human research addressing DGAT‐1 inhibition. The postprandial triglyceride concentration was massively lowered with no adverse, cardiovascular‐related complications reported. However up to 43 % of the study participants experienced gastrointestinal issues including, nausea, diarrhea and vomiting. Adverse events were lowered, if the total fat content of the meal and the dosage of the drugs were decreased.^13^

### 1.3 Cases reported in the literature to this date

Up to now, only a few case reports of DGAT1 deficiency have been published. Table S1 summarizes the different manifestations and outcomes of known patients.

## 2 CASE REPORT

Until the time of admission, the child was fully breast fed. The mother noticed large volumes of stool every 2 hours at the age of 4 weeks. The stool of the child showed low pancreas elastase concentrations (44 µg/g; reference range: >200 µg/g). A combination of infusions and oral administration of exogenous digestive enzymes only slightly improved the symptoms. When the patient was admitted to the hospital, he was 39 days old and had a profound dystrophy with a body weight of 3.47 kg at a length of 52 cm (BMI = 12.8 kg/m2 <1st percentile).

Laboratory tests revealed a normal acid‐base balance, high parathyroid hormone (131 µg /ml; reference range: 15.6–65 µg /ml) and low 25‐OH‐Vitamin D3 (12 ng/ml; deficiency classified as <10 ng/ml, inadequate supply classified as 10–30 ng/ml). Other fat‐soluble vitamins like A (<0.1 mg/l) and E (<5 mg/l) were shown to be also very low. Zinc was also below the physiological range at 536 µg/l (reference range: 750‐1400 µg/l). Blood counts and further clinical chemistry analysis of serum showed no significant abnormalities.

Because of insufficient weight gain, the child started to get total parenteral nutrition. This stopped the persistence of diarrhea.

Molecular genetic analysis (Figure S1) showed homozygosity for c.140del C; p.(Pro47Glnfs*20) in the DGAT1 gene, both parents were heterozygous.

After diagnosis at the age of 16 weeks, the patient was put on a fat‐free infant formular diet supplemented with caprylic acid (C8) medium chain triglyceride (MCT) oil. Total MCT oil dosage was about 1 ml per kg body weight per day. Vitamin D and K1 supplementation were given.

At the age of 20 weeks, the patient had a weight of 6410g (10th percentile) and length of 62 cm (<10th percentile) according to “KIGGS percentile curves”, showing improved weight and length gain compared to the time before.

**Figure 1:**
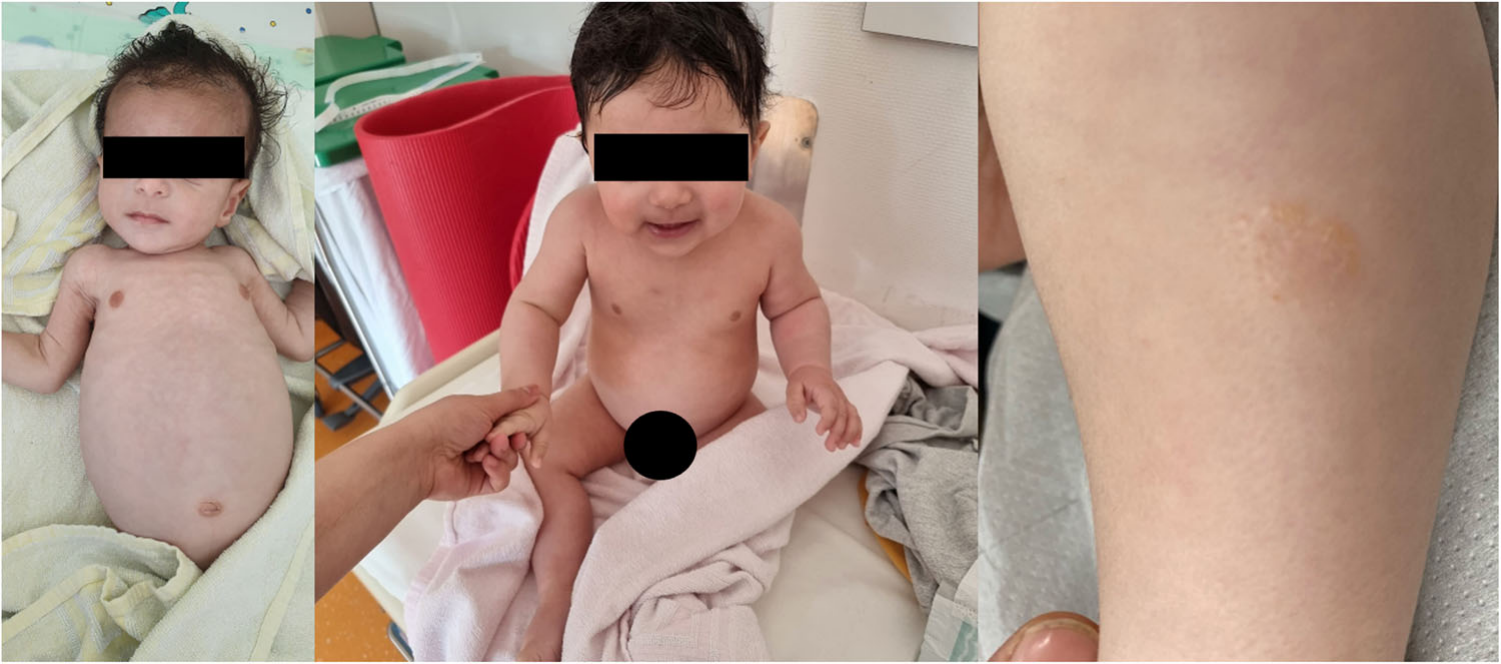
Left: Patient at the age of 6 months showing severe dystrophy, Middle: At the age of 9 months after initiating treatment, Right: Skin lesion of the patient caused by essential fatty acid deficiency

Essential fatty acids decreased to very low levels and led to multiple skin lesions (Figure 1).

**Figure 2:**
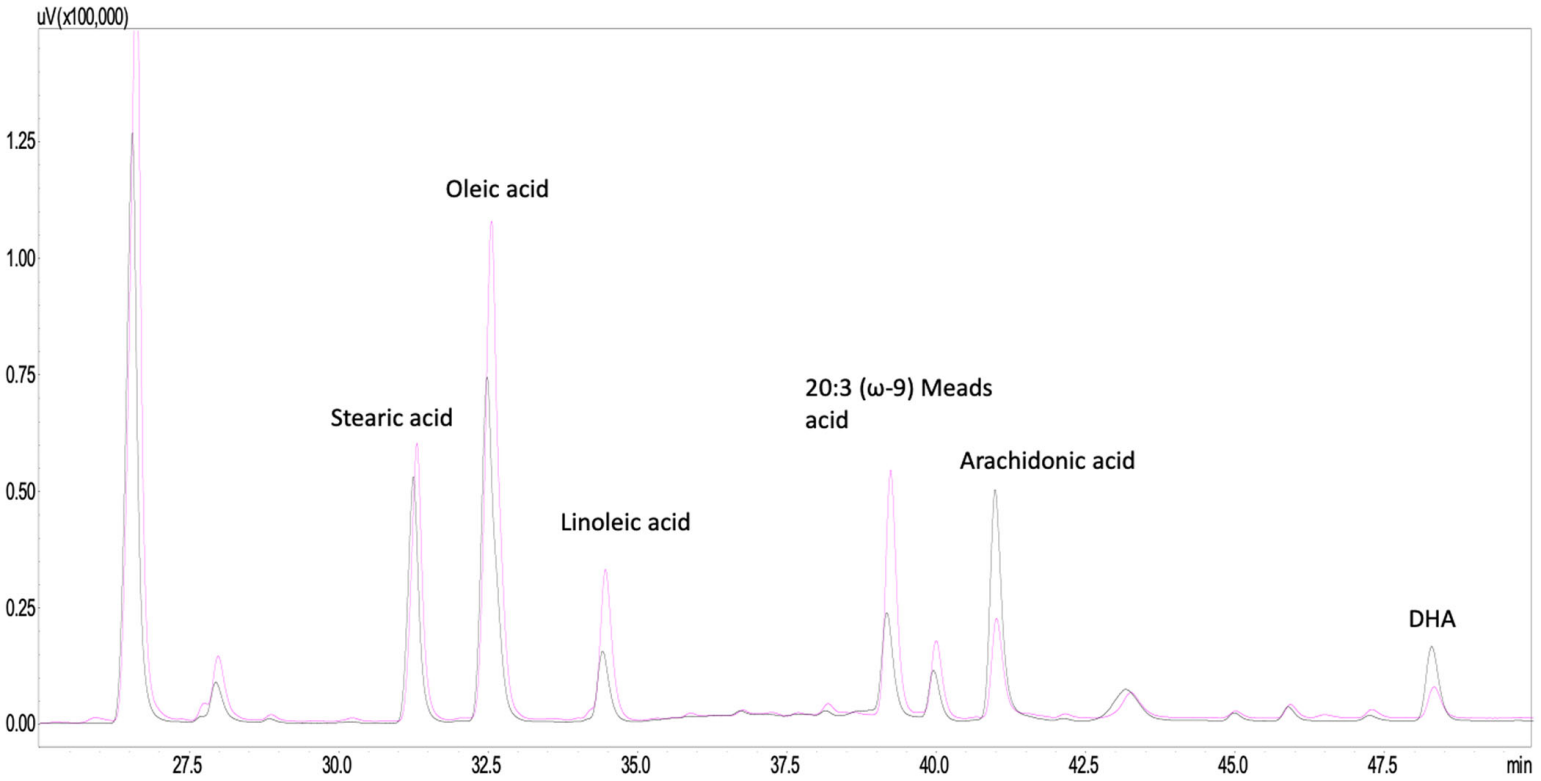
Chromatograms of fatty acid profile of the phoshpholipid compositions in serum pre (pink) and post (black) treatment with omega 3/6 supplement. DHA, docosahexaenoic acid

Analysis revealed low linoleic acid (6.3 %, reference range: 10.3–21.8%), low arachidonic acid (4.4%, reference range: 6.4–12.2%) and low docosahexaenoic acid (DHA) (1.4%, reference range: 1.68–6.6%) concentrations in the blood. Blood concentrations of palmitoleic acid (2.8%, reference range: 0.3–1.6%), oleic acid (22.7%, reference range: 11.3 – 16.6%), Mead's acid (9.49%, reference range: 0.09–1.36%) and eicosapentaenoic acid (EPA) (1.77%, reference range: 0.41–1.72%) were classified as high. Alpha‐linoleic acid was within range (0.06%, reference range: 0.02–0.37%). Chromatograms of fatty acid profile are shown in Figure 2.

The patient continued to gain weight. At the age of 32 weeks, body weight was 10 kg (80th percentile according to WHO, see Figure S3). In order to treat the essential fatty acid deficiency, an Omega 3/6 supplement was given once daily containing 100 mg of arachidonic acid and 200 mg of DHA per serving. The changes in lipid levels before and after the treatment are shown in Table S2. In addition to the fat free formula, the patient started to eat some fruit and vegetables.

## 3 RESULTS

After treatment initiation, the patient went into full remission of all gastrointestinal problems. The patient started to gain weight and finally was within the range of the anticipated weight percentiles (Figure S2).

One year after birth, the child was again experiencing persistent diarrhea. Investigations revealed that the patient was eating a total amount of 65.5 g of fructose in a given day, leading to diarrhea caused by fructose malabsorption. Daily fructose consumption exceeding 25 g is sufficient to induce fructose malabsorption in healthy adults, thus already causing gastrointestinal issues. The effect was even higher (two‐thirds of the test subjects), if the total daily amount of fructose exceeded 50 g.^14^ It is important to note that all study participants were healthy adults, indicating the dosage for a 2‐year‐old child with a weight of 13 kg to cause gastrointestinal problems might be much lower.

## 4 DISCUSSION

We presented a case of a patient with DGAT‐1 deficiency leading to congenital diarrhea and failure‐to‐thrive. It was possible to put the patient into full remission via dietary modifications thus achieving the anticipated growth and weight parameters. During the first year of life we relied exclusively on a fat‐free‐infant formula with the following diet mostly consisting of large amounts of carbohydrate, nearly no fat and adequate amounts of protein. To compensate for a potential protein deficiency, dietary protein supplements (free of fat) were used. We used a list which ranked foods from lowest to highest content of fat, so the caretaker of the child was able to choose which foods where suitable for the specific diet. Depending on individual tolerability a diet plan should be advised which consists of food containing less than 1g of fat per 100g.

MCTs are absorbed via epithelial cells, bound to albumin and immediately transported to the liver via the portal vein. The absorption is possible both with and without cleavage, while the primary oxidation happens in the liver.^15^ This could be an explanation for the possibility of the patient to absorb MCT, as he experienced no gastrointestinal problems to C8‐MCT administration, which is the novelty presented in our case compared to the published literature until this day. A clinical study investigating the impact of both MCT and LCT – based baby formula on postprandial chylomicron buildup showed different results. Although the absolute triglyceride synthesis after MCT ingestion was low, the amount of MCT incorporated into chylomicron triglycerides was significant.^16^ Since the incorporation of MCT into chylomicrons is chain length dependent, C8 oil was chosen over the more common C8/C10 mixtures.^17^

Both long‐chain‐ and essential‐fatty‐acids are mostly reacylated into triglycerides in the enterocyte, where they get assembled into chylomicrons for transportation to the lymph's. The triglyceride synthesis follows and is mostly regulated via DGAT‐1. But there exists an alternative pathway involving the conversion of glycerol‐3‐phosphatase via phosphatidic acid to diacylglycerol and later on to triglycerides via multiple enzymes.^18^

Further research is required to analyze how specific fatty acids impact chylomicrons synthesis.

The patient showed very low alpha‐linoleic acid concentrations in the blood before essential fatty acids were supplemented, whereas EPA and DHA were in the reference range. Literature indicates alpha‐linoleic acid is a limited source of longer omega‐3 fats for the human organism, with women being a superior converter of alpha‐linoleic acid to EPA and DHA, and an improvement of serum DHA levels for both genders is only possible with DHA supplementation.^19^, ^20^, ^21^

## 5 CONCLUSION

DGAT‐1 deficiency is a rare genetic disease, which causes severe gastrointestinal issues, potentially life threatening in early life. Treatment is possible via a fat‐free diet combined with specific, dietary supplementation which lead to normalization of weight percentiles. C8‐MCT administration did not cause any issues. To prevent essential fatty acid deficiency, a daily supplement should be used, which also does not seem to cause gastrointestinal issues.

Further research is required to investigate the different metabolizations of fatty acids to explain the different chylomicron buildups and further improve the treatment of patients diagnosed with DGAT‐1 deficiency.

## CONFLICT OF INTEREST STATEMENT

The authors declare no conflicts of interest.

## ETHICS STATEMENT

Due to working with very rare (metabolic) diseases, we have a universal ethics committee approval. Written consent was obtained from the patient's parent.

## Supporting information

Figure S1: Sanger sequencing revealed patient (A) carrying a homozygous mutation, father (B) and mother (C) being heterozygous for this mutation.

Figure S2: Growth curve of the patient (KIGGS).

Figure S3: Growth curve of the patient (WHO).

Table S1: Comparison of symptoms, treatments and outcomes in patients diagnosed with DGAT‐1 deficiency. Table S2: Changes in fatty acid concentrations after administration of omega 3/6 supplement.

## References

[1] Haas JT , Winter HS , Lim E , et al. DGAT1 mutation is linked to a congenital diarrheal disorder. J Clin Invest. 2012;122(12):4680‐4684. 23114594 10.1172/JCI64873 PMC3533555

[2] Amin NB , Saxena AR , Somayaji V , Dullea R . Inhibition of diacylglycerol acyltransferase 2 versus diacylglycerol acyltransferase 1: potential therapeutic implications of pharmacology. Clin Ther. 2023;45(1):55‐70. 36690550 10.1016/j.clinthera.2022.12.008

[3] Deolet E , Callewaert B , Geldof J , et al. Apoptotic enteropathy, gluten intolerance, and IBD‐like inflammation associated with lipotoxicity in DGAT1 deficiency–related diarrhea: a case report of a 17‐year‐old patient and literature review. Virchows Arch. 2022;481(5):785‐791. 35763111 10.1007/s00428-022-03365-w

[4] Gluchowski NL , Chitraju C , Picoraro JA , et al. Identification and characterization of a novel DGAT1 missense mutation associated with congenital diarrhea. J Lipid Res. 2017;58(6):1230‐1237. 28373485 10.1194/jlr.P075119 PMC5454518

[5] van Rijn JM , Ardy RC , Kuloğlu Z , et al. Intestinal failure and aberrant lipid metabolism in patients with DGAT1 deficiency. Gastroenterology. 2018;155(1):130‐143.e15. 29604290 10.1053/j.gastro.2018.03.040 PMC6058035

[6] Chen HC , Smith SJ , Ladha Z , et al. Increased insulin and leptin sensitivity in mice lacking acyl CoA: diacylglycerol acyltransferase 1. J Clin Invest. 2002;109(8):1049‐1055. 11956242 10.1172/JCI14672 PMC150948

[7] Smith SJ , Cases S , Jensen DR , et al. Obesity resistance and multiple mechanisms of triglyceride synthesis in mice lacking Dgat. Nat Genet. 2000;25(1):87‐90. 10802663 10.1038/75651

[8] Streeper RS , Grueter CA , Salomonis N , et al. Deficiency of the lipid synthesis enzyme, DGAT1, extends longevity in mice. Aging (Albany NY). 2012;4(1):13. 22291164 10.18632/aging.100424 PMC3292902

[9] Roe ND , Handzlik MK , Li T , Tian R . The role of diacylglycerol acyltransferase (DGAT) 1 and 2 in cardiac metabolism and function. Sci Rep. 2018;8(1):4983. 29563512 10.1038/s41598-018-23223-7 PMC5862879

[10] Ables GP , Yang KJZ , Vogel S , et al. Intestinal DGAT1 deficiency reduces postprandial triglyceride and retinyl ester excursions by inhibiting chylomicron secretion and delaying gastric emptying. J Lipid Res. 2012;53(11):2364‐2379. 22911105 10.1194/jlr.M029041 PMC3466005

[11] Bang‐Andersen B , Ruhland T , Jørgensen M , et al. Discovery of 1‐[2‐(2,4‐dimethylphenylsulfanyl)phenyl]piperazine (Lu AA21004): a novel multimodal compound for the treatment of major depressive disorder. J Med Chem. 2011;54(9):3206‐3221. 10.1021/jm101459g 21486038

[12] Denison H , Nilsson C , Löfgren L , et al. Diacylglycerol acyltransferase 1 inhibition with AZD7687 alters lipid handling and hormone secretion in the gut with intolerable side effects: a randomized clinical trial. Diabetes Obes Metab. 2014;16(4):334‐343. 24118885 10.1111/dom.12221

[13] Devita RJ , Pinto S . Current status of the research and development of diacylglycerolO‐acyltransferase 1 (DGAT1) inhibitors: miniperspective. J Med Chem. 2013;56(24):9820‐9825. 23919406 10.1021/jm4007033

[14] Beyer PL , Caviar EM , McCallum RW . Fructose intake at current levels in the United States may cause gastrointestinal distress in normal adults. J Am Diet Assoc. 2005;105(10):1559‐1566. 16183355 10.1016/j.jada.2005.07.002

[15] Hermann H . Biochemie‐Klassiker: Kurzes Lehrbuch der Biochemie für Mediziner und Naturwissenschaftler. Von Peter Karlson. Georg Thieme Verlag, Stuttgart 1984. 12. völlig neu bearbeitete Auflage. XII, 452 S., 177 Abb., 37 Tab., 323 Formelbilder und Schemata, kart. DM 49,80. ISBN 3‐13‐357812‐X. Nachr Chem Tech Lab. 1986;34(2):166‐167.

[16] Swift L , Hill J , Peters J , Greene H . Medium‐chain fatty acids: evidence for incorporation into chylomicron triglycerides in humans. Am J Clin Nutr. 1990;52(5):834‐836. 2239759 10.1093/ajcn/52.5.834

[17] Lee DS , Hashim SA , Van Itallie TB . Effect of long chain triglyceride on chylous transport of medium chain fatty acids. Am J Physiol. 1968;214(2):294‐299. 10.1152/ajplegacy.1968.214.2.294 5635872

[18] Minich DM , Vonk RJ , Verkade HJ . Intestinal absorption of essential fatty acids under physiological and essential fatty acid‐deficient conditions. J Lipid Res. 1997;38(9):1709‐1721. 9323581

[19] Burdge GC , Calder PC . Conversion of α‐linolenic acid to longer‐chain polyunsaturated fatty acids in human adults. Reprod Nutr Dev. 2005;45(5):581‐597. 16188209 10.1051/rnd:2005047

[20] Burdge GC , Wootton SA . Conversion of α‐linolenic acid to eicosapentaenoic, docosapentaenoic and docosahexaenoic acids in young women. Br J Nutr. 2002;88(4):411‐420. https://www.cambridge.org/core/journals/british-journal-of-nutrition/article/conversion-of-linolenic-acid-to-eicosapentaenoic-docosapentaenoic-and-docosahexaenoic-acids-in-young-women/2B640958BD4A0061593384DF076DBC74 12323090 10.1079/BJN2002689

[21] Brenna JT , Salem N , Sinclair AJ , Cunnane SC . α‐Linolenic acid supplementation and conversion to n‐3 long‐chain polyunsaturated fatty acids in humans. Prostaglandins Leukot Essent Fatty Acids. 2009;80(2‐3):85‐91. 19269799 10.1016/j.plefa.2009.01.004

