# Supplementary figures and images for: DGAT‐1 deficiency: Congenital diarrhea and dietary treatment
Source: JPGN Rep. 2025 Apr 9;6(2):121–5. doi: 10.1002/jpr3.70016 (PMC12078034; doi:10.1002/jpr3.70016)

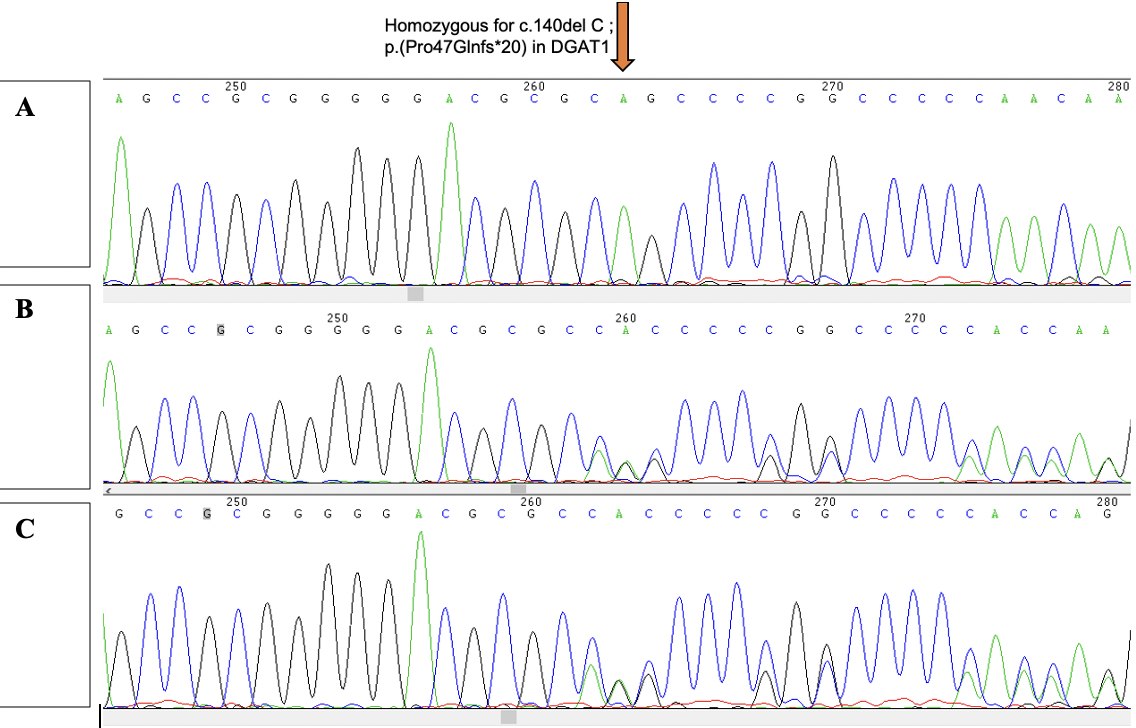

Supplement: Supplementary file 1 — Figure S1: Sanger sequencing revealed patient (A) carrying a homozygous mutation, father (B) and mother (C) being heterozygous for this mutation. [file JPR3-6-121-s004.tiff]

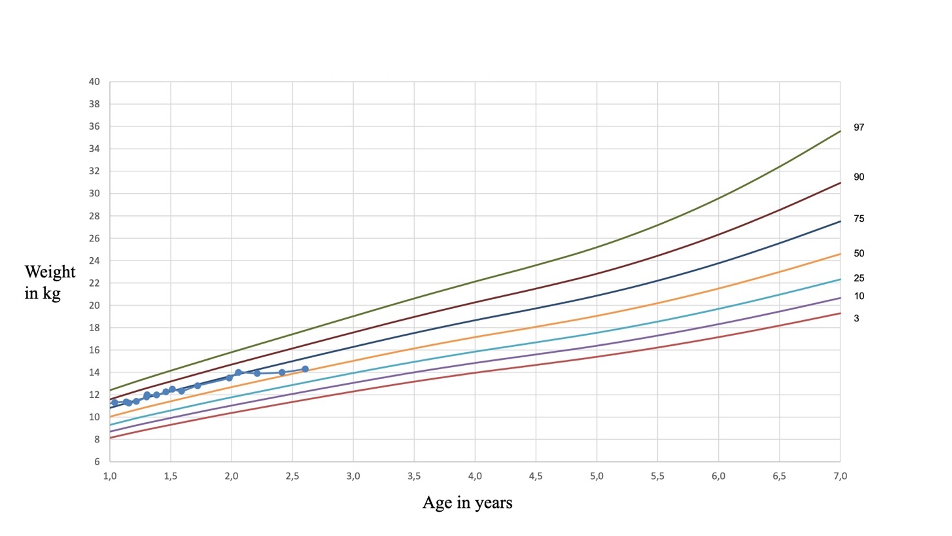

Supplement: Supplementary file 2 — Figure S2: Growth curve of the patient (KIGGS). [file JPR3-6-121-s001.tiff]

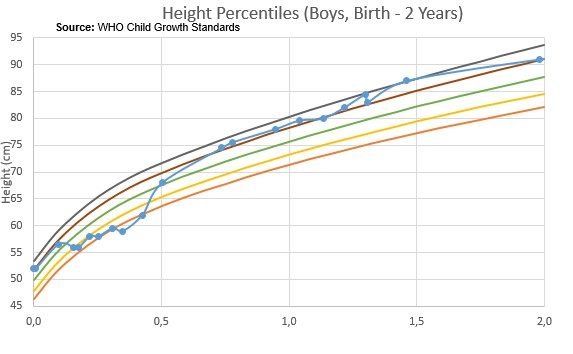

Supplement: Supplementary file 3 — Figure S3: Growth curve of the patient (WHO). [file JPR3-6-121-s003.tiff]
