# Supplementary material for: DGAT‐1 deficiency: Congenital diarrhea and dietary treatment
Source: JPGN Rep. 2025 Apr 9;6(2):121–5. doi: 10.1002/jpr3.70016 (PMC12078034; doi:10.1002/jpr3.70016)
Supplement: Supplementary file 4 — Table S1: Comparison of symptoms, treatments and outcomes in patients diagnosed with DGAT‐1 deficiency. Table S2: Changes in fatty acid concentrations after administration of omega 3/6 supplement. [file JPR3-6-121-s002.docx]

| Sex | Onset | Symptoms | Treatment | Outcome | Source |
| --- | --- | --- | --- | --- | --- |
| Female | 6 weeks | Severe congenital diarrhea, protein-losing enteropathy, damaged epithelium | Discontinuation  of enteral feeding, reintroduction of enteral feeding at the age of 3, oral feeding of strict low-fat diet with supplemented essential fatty acids | Development of IBD-like inflammation and celiac disease, later manifestation of anorexia nervosa | (3) |
| Male (twins) | Shortly after birth | Watery diarrhea and failure to thrive, protein-losing enteropathy, only one sibling severely affected and needed hospitalization | Very low-fat enteral diet, red blood cell transfusion and intravenous immunoglobulin administration | Improvement of the patient’s growth abnormalities | (7) |
| Male, Female  (Siblings) | Female:  3 days after birth | Female: vomiting, colicky pain, watery diarrhea. Protein-losing enteropathy, hyperlipidemia  Male: non-bloody, watery diarrhea, dehydration, metabolic acidosis and hyponatremia, increased alpha-1-antitrypsin, protein-losing enteropathy | Female: oral rehydration formula and switch to soy-based formula, parenteral nutrition and albumin infusions, tube feeding with amino acid-based formula containing maltodextrin and MCT  Male: Intravenous albumin injections, amino acid based formula and parenteral nutrition, cholestyramine | Female: Below first percentile for weight at age of 14 months, died at the age of 17 months due to complications of malnutrition and sepsis  Male: Weight gain and no longer loosing protein in his stool at the age of 10 months, diarrhea improved, fasting lipid levels decreased after cholestyramine administration, thriving while eating an unrestricted diet at the age of 46 months | (8) |
| Male | Two weeks after birth | Persistent diarrhea and failure to thrive, IgG < 108 mg/dl, Hypoalbuminemia, elevated alpha-1-antitrypsin | Intravenous immunoglobulin injections, exclusively low-fat formula | Remission and normal growth parameters | (9) |

**Supp Tab. 1: Comparison of symptoms, treatments and outcomes in patients diagnosed with** **DGAT-1 deficiency**

| Fatty Acid concentration (GC analysis) | Before Key Omega administration | After Key Omega administration | Reference Range |
| --- | --- | --- | --- |
| Palmitoleic Acid | 2.8 % | 2.2 % | 0.3 – 1.6 % |
| Oleic Acid | 22.7 | 20.8 % | 11.3 – 16.6 % |
| Linoleic Acid | 6.3 | 3.8 % | 10.3 – 21.8 % |
| Alpha-Linoleic Acid | 0.12 | 0.04 % | 0.02 -0.37 % |
| Meadsche Acid | 9.49 | 4.98 % | 0.09 – 1.36 % |
| Arachidonic Acid | 4.4 | 12.7 % | 6.4 – 12.2 % |
| EPA | 1.77 | 3.59 % | 0.41 – 1.72 % |
| DHA | 1.4 | 4.26 % | 1.68 – 6.6 % |

**Suppl Tab. 2: Changes in fatty acid concentration after administration of Key Omega**
